# Probing the electronic and spintronic properties of buried interfaces by extremely low energy photoemission spectroscopy

**DOI:** 10.1038/srep08537

**Published:** 2015-02-23

**Authors:** Roman Fetzer, Benjamin Stadtmüller, Yusuke Ohdaira, Hiroshi Naganuma, Mikihiko Oogane, Yasuo Ando, Tomoyuki Taira, Tetsuya Uemura, Masafumi Yamamoto, Martin Aeschlimann, Mirko Cinchetti

**Affiliations:** 1 Department of Physics and Research Center OPTIMAS, University of Kaiserslautern, Erwin-Schrödinger Str. 46, 67663 Kaiserslautern, Germany; 2 Department of Applied Physics, Graduate School of Engineering, Tohoku University, aoba-yama 6-6-05, Sendai 980-8579, Japan; 3 Division of Electronics for Informatics, Hokkaido University, Kita 14 Nishi 9, Sapporo 060-0814, Japan

## Abstract

Ultraviolet photoemission spectroscopy (UPS) is a powerful tool to study the electronic spin and symmetry features at both surfaces and interfaces to ultrathin top layers. However, the very low mean free path of the photoelectrons usually prevents a direct access to the properties of buried interfaces. The latter are of particular interest since they crucially influence the performance of spintronic devices like magnetic tunnel junctions (MTJs). Here, we introduce spin-resolved extremely low energy photoemission spectroscopy (ELEPS) to provide a powerful way for overcoming this limitation. We apply ELEPS to the interface formed between the half-metallic Heusler compound Co_2_MnSi and the insulator MgO, prepared as in state-of-the-art Co_2_MnSi/MgO-based MTJs. The high accordance between the spintronic fingerprint of the free Co_2_MnSi surface and the Co_2_MnSi/MgO interface buried below up to 4 nm MgO provides clear evidence for the high interface sensitivity of ELEPS to buried interfaces. Although the absolute values of the interface spin polarization are well below 100%, the now accessible spin- and symmetry-resolved wave functions are in line with the predicted existence of non-collinear spin moments at the Co2MnSi/MgO interface, one of the mechanisms evoked to explain the controversially discussed performance loss of Heusler-based MTJs at room temperature.

Magnetic tunnel junctions (MTJs) are essential parts of many magnetic storage devices. Typically, they can be found in magnetic hard-disks as high sensitivity read-heads or in non-volatile and fast-access magnetic random access memory elements as basic building block^1^,^2^. These structures consist of two ferromagnetic electrodes separated only by a few-nanometer thin insulating layer. The relative magnetization direction of the two ferromagnetic metals (FM), i.e., the electrodes, is directly reflected in the tunneling current across the MTJ. While a parallel orientation of the magnetization in the ferromagnetic films results in a high tunneling current, an anti-parallel orientation leads to a significantly lower tunneling current. Therefore, MTJs show the distinct behavior of a two level system which can be switched by an external magnetic field and hence be employed in a variety of logical circuits.

The applicability of MTJs in storage devices crucially depends on the difference between the resistance of these two states, the so called tunnel magneto-resistance ratio, which is a direct result of the intrinsic properties of the MTJs. In particular, to improve the performance of the MTJs, the fundamental electronic properties of the buried ferromagnetic metal/insulator interface have to be revealed. The importance of the interface results from the fact that only electrons from the outermost FM layers can participate to the tunneling process^3^,^4^. For high efficiency MTJs using epitaxial MgO barriers as insulating layer, the spin-dependent tunneling properties crucially depend not only on the interface spin polarization, but also on the wave function symmetry of the electronic states near *E_F_* at that buried FM/MgO interface^5^,^6^. Up to now, spin-resolved ultraviolet photoemission spectroscopy (UPS) is the only existing method capable of revealing both the electronic spin and symmetry properties at interfaces^7^. However, sample structures are constrained to FM substrates with ultrathin insulator top layers of 1–2 ML (i.e. less than 0.5 nm)^8^, whereas direct access to interfaces buried below thicker insulator layers comparable to actual devices (i.e. 1–2 nm) is experimentally very challenging due to the very low mean free path of electrons in materials^9^. This is quite a drawback of UPS, since the interface properties themselves depend on the thickness of the insulating layer^10^.

Recently, significant process in the development of high brilliant photon sources (3rd generation synchrotron light sources) allowed to increase the escape depth of electrons to a few nanometers by using hard X-ray photons of several hundreds of electron volts (HAXPES) which resulted in the first studies of the electronic structure of buried interfaces^11^. However, the low cross section for the photoemission process in the HAXPES experiment (especially for electronic states close to the Fermi level) is still a huge challenge when studying the corresponding spin properties of buried interfaces. In addition, the experiment becomes distinctively bulk-sensitive. Consequently, alternative approaches are essential to extend our knowledge of buried interfaces in electronic and spintronic assemblies.

Here, we demonstrate how spin-resolved extremely low energy photoemission spectroscopy (ELEPS) performed with photon energy of 5.9 eV can be used to study the electronic and spintronic properties of buried interfaces between a ferromagnetic metal (FM) and an insulator. As FM/insulator structure, we choose materials suited for high performance MTJs. One of the most promising ferromagnetic materials in this context are fully spin polarized half-metallic ferromagnets such as the cobalt-based full Heusler compound Co_2_MnSi. This material has a high Curie Temperature (985 K)^12^ and a predicted minority band gap around the Fermi energy (*E_F_*) with a width of 0.4–0.8 eV^13^,^14^, that should lead to a full spin polarization at *E_F_*. Epitaxial MgO, commonly used as insulating material in magnetic tunnel junctions due to its symmetry-filtering properties^5^,^6^, is grown on top of the Heusler complex. The studied Co_2_MnSi/MgO interfaces have been deposited using the exact same parameters as in the production of state-of-the-art Co_2_MnSi-based MTJs. These devices show superior performance, with TMR ratios between 750% and 1135% at cryogenic temperatures^15^,^16^. In our ELEPS experiments we find that the properties of such Co_2_MnSi/MgO interfaces are accessible for MgO overlayers with thickness up to 4 nm (20 ML), making ELEPS a unique method for the non-destructive characterization of buried spintronics interfaces, as found in state-of-the-art Co_2_MnSi/MgO-based MTJs^17^. Taking advantage of such interface sensitivity we report a room-temperature interface spin polarization of ≈40% at the Fermi energy, in striking contrast with Ref. 18, where a nearly full spin polarization (93%) is reported for the free Co_2_MnSi surface. In principle, there are two possible explanations for the reduced spin polarization: (i) temperature effects, or (ii) effects inherent to the photoemission process^18^.

Regarding the temperature-related effects (i), we recall that although Co_2_MnSi-based MTJs show strongly enhanced tunnel magneto-resistance ratios^19^ at liquid helium temperature compared to MTJs based on conventional 3*d* ferromagnetic materials^20^, the superiority is lost at room temperature^15^. This behavior is commonly ascribed to a strong temperature-dependence of the spin polarization at the Co_2_MnSi/MgO interface, but a clear microscopic understanding is still lacking. In particular, two different main mechanisms have been proposed, schematically depicted in Fig. 1 (a). The first mechanism is the formation of additional minority band gap states or the shifting of existing minority states inside the band gap. These processes can arise either from peculiarities of the magnetic sublattices^21^, from the existence of non-quasiparticle states^22^, from spin wave excitations^23^ or from strong hybridization dependence on temperature^24^. At room temperature, all such processes lead to finite minority spectral contributions near *E_F_* that will differ in energy dispersion and symmetry compared to the majority states at same binding energy. The second mechanism is the formation of non-collinear spin moments inside the half-metal bulk as well as at the interface^24^,^25^,^26^, leading to a mirroring of majority states into the minority channel and hence to minority gap states with the same wave function symmetry as the majority states.

Regarding the photoemission-related mechanisms (ii), it was recently suggested^18^ that correlation effects in the photoemission process together with the finite energy resolution of standard spin-detectors can cause a reduction of the detected spin polarization from the nominal 100% down to ≈50%.

In order to distinguish between all the possible contributions resulting in a lowering of the spin-polarization at room temperature, we will make use of the further possibility of ELEPS to resolve the relevant wave function symmetries (Δ_1_ and Δ_5_), demonstrating the high potential of ELEPS for the non-destructive characterization of the spin properties of buried FM/insulator spintronics interfaces.

## Results

### The Co_2_MnSi(100) free surface

As a basis to understand the results on the Co_2_MnSi/MgO interface, we start discussing here the results obtained from the bare Co_2_MnSi(100) surface. Note that the photoemission experiments from the free surface have been performed after those on the interface; to do so we removed the MgO overlayer by Ar^+^ ion sputtering (see Methods).

In general, the photoemission signal obtained in our experiments stems mainly from initial electronic states near *E_F_* with either Δ_5_ or Δ_1_ wavefunction symmetry, located within the Γ – *X* part of the Co_2_MnSi Brillouin zone^27^ (see Methods). Please note that exactly these states contribute almost exclusively to the tunneling current in the respective MTJ devices^5^,^28^. Changing the light polarization furthermore allows us to distinguish between the two wave function symmetries, as linearly s-polarized light excites only Δ_5_ states, while p-polarized light probes additionally Δ_1_ states^7^,^27^. This feature is one huge advantage (amongst others) of spin-resolved photoemission spectroscopy compared to further spin-sensitive experiments like Point contact Andreev reflection^29^ or the Meservey & Tedrow technique^3^.

**Figure 1:**
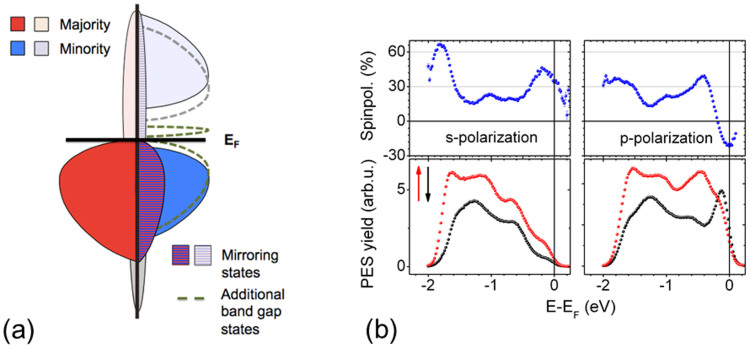
The Heusler compound Co_2_MnSi. (a) Representation of the two main mechanisms leading to thermally induced loss of spin-polarization close to *E_F_*: (i) Mirroring of majority states in the minority channel due to non-collinear spin moments at the surface/interface, and (ii) energetic shifts of existing states or formation of new minority states. (b) Majority (red) and minority (black) electron photoemission spectra of the bare Co_2_MnSi(100) surface (lower panel) and deduced spin polarization (upper panel), measured by spin-resolved ELEPS (*hν* = 5.9 *eV*) with s-polarized (left) and p-polarized laser light (right).

The lower panels of Fig. 1 (b) show the spin-resolved ELEPS spectra measured with s-polarized (left) and p-polarized laser light (right), while the upper panels exhibit the respective spin polarization. The latter one is calculated from the experimentally obtained majority and minority spectra (*N*_↑_ and *N*_↓_, respectively) as 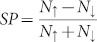. In this section as well as in the further ones, we will focus on the features found close the Fermi level, the energy region where spin-polarized charge transport takes place in spintronics devices. In case of s-polarization, we find a rather low photoemission yield directly at *E_F_* (additionally cut by the Fermi-Dirac distribution), getting more intense for binding energies higher then 0.4 eV. This is expected from bulk band structure calculations, which predict Δ_1_ and Δ_5_ bands along the Γ – *X* direction with strong dispersion around the Fermi energy and becoming rather flat at approx. 0.5 eV binding energy^12^,^28^. The respective spin polarization shows a maximum at *E* − *E_F_* = −0.2 *eV* with a value of +45%. Both spectra and spin-polarization change dramatically at *E_F_* when p-polarized light is used for excitation. In this case the minority channel exhibits a prominent peak directly at *E_F_*, leading to a distinct negative spin polarization of −20%. This peak can be ascribed to a minority surface state with Δ_1_-like symmetry for three reasons: (i) it can only be excited by p-polarized light^30^; (ii) several theoretical works^27^,^31^ predict minority surface states for Co_2_MnSi(001) and have been already confirmed experimentally for off-stoichiometric Co_2_MnSi in our previous work^27^; (iii) the state vanishes when the surface is covered by MgO, as described in detail in the following section.

The values of the spin polarization at *E_F_* (45% for s-polarized light and −20% for p-polarized light) are in apparent disagreement with the value of 93% recently measured by spin-resolved UPS^18^. We first recall that the photoemission calculations reported in Ref. 18 have shown that even considering a fully spin polarized Co_2_MnSi sample, the broadening due to electronic correlations and the experimental energy resolution reduce the measured spin polarization to values below 55% if only bulk photoemission transitions are considered. This value is indeed very close to the spin polarization measured by ELEPS with s-polarized light, which is mostly sensitive to bulk electric states. In the UPS measurements of Ref. 18, the used photon energy of 21.2 eV excites further surface resonances in the majority channel, leading to the measured high spin polarization. Indeed the majority ELEPS spectra measured with p-polarized light, which is additionally sensitive to surface features, show a higher photoemission yield near *E_F_* than the spectra measured with s-polarized light. However, in ELEPS the 5.9 eV photons induce a resonant excitation of a pure surface state in the minority channel, as clearly visible in the minority spectra in Fig. 1 (b) (p-polarization). This resonant excitation reduces the spin polarization down to negative values instead of increasing it up to 93% as in UPS. It is thus possible to reconcile our ELEPS results to previous UPS measurements^18^. However, we add here that the reduced spin polarization of the Co_2_MnSi surface might also have thermal origin, as discussed in detail in the next section.

### The buried Co_2_MnSi/MgO interface

**Figure 2:**
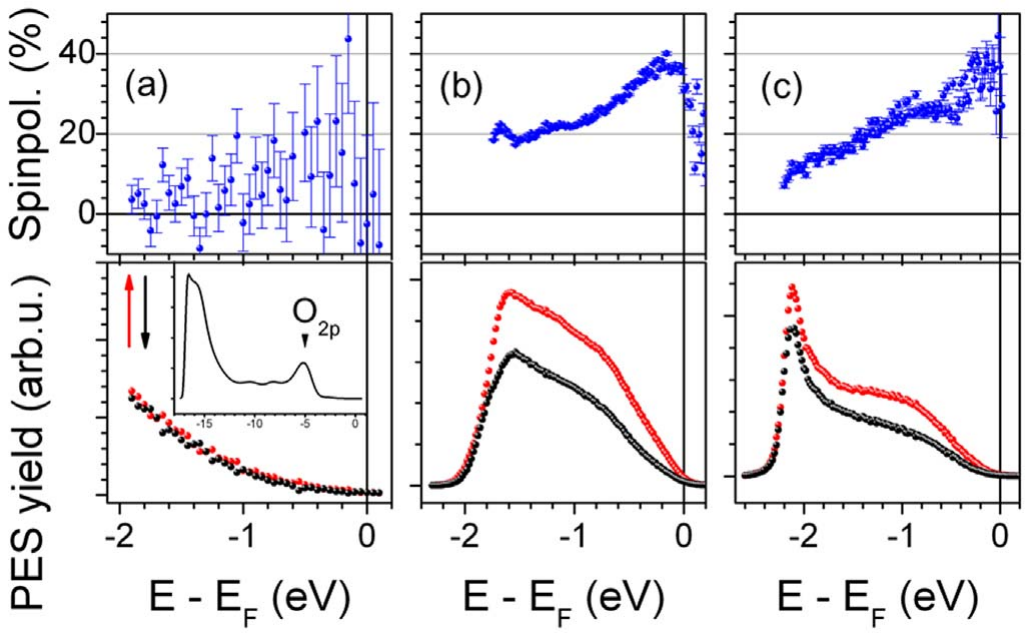
Photoelectron spectroscopy of the Co_2_MnSi/MgO interface. Majority (red) and minority (black) electron photoemission spectra of the Co_2_MnSi/MgO(10 ML) sample (lower panel) and deduced spin polarization (upper panel), measured with spin-resolved UPS (h*ν* = 21.2 eV) (a) and spin-resolved ELEPS (h*ν* = 5.9 eV) with p-polarized light (b). (c) same as (b), but for the Co_2_MnSi/MgO(20 ML) sample. Inset in (a): full UPS spectrum of the Co_2_MnSi/MgO(10 ML) sample with spectral feature from the oxygen 2p peak. (Peak maximum at *E* − *E_F_* = −5 *eV*, peak onset at *E* − *E_F_* = −4 *eV*.)

Let us now turn to the results obtained for the Co_2_MnSi/MgO(10 ML) system (1 ML MgO equals 0.21 nm). Figure 2 (a) shows conventional spin-resolved UPS spectra measured with a photon energy of h*ν* = 21.2 eV (bottom panel) and the corresponding spin-polarization (upper panel). In these spectra almost no photoemission signal is detected close to *E_F_*. For lower binding energies a distinct feature centered at *E* − *E_F_* = −5 *eV* stemming from the oxygen 2p states of MgO^11^,^32^ is observed (inset of Fig. 2 (a)). As expected, the UPS study performed with h*ν* = 21.2 eV gives mainly spectroscopic information about the MgO surface and not about the interface to Co_2_MnSi, since the MgO top layer thickness (ca. 2 nm) is larger than the electronic mean free path in MgO, the latter being ≤1 nm for photon energies between 20 and 500 eV (NIST Electron Inelastic-Mean-Free-Path Database). Further confirmation is given by the clearly visible MgO band gap ranging from *E_F_* down to 4 eV binding energy, the onset of the MgO valence band.

The spin-resolved ELEPS spectra (measured with h*ν* = 5.9 eV) show a completely different behavior: Most remarkable, the spectra for p- (shown in Fig. 2 (b)) and s-polarized laser light (not shown here) resemble the ones obtained at the free Co_2_MnSi surface using s-polarized laser light (c.f. Fig. 1(b)). Even the inferred spin polarization shows high similarity to the free Co_2_MnSi surface (in case of s-polarized light) with a maximum value of 40% in vicinity of the Fermi energy, dropping down to roughly 20% at higher binding energies. In addition, the total photoemission yield of the Co_2_MnSi/MgO(10 ML) structure is not lowered distinctively compared to the free Co_2_MnSi surface. These findings prove that - in contrast to conventional UPS - ELEPS allows to determine the spin-polarization of the buried interface throughout a MgO thickness of 10 ML. Even more surprising, this observation still holds for thicker MgO films as demonstrated by the spin-resolved ELEPS spectra measured from the Co_2_MnSi/MgO(20 ML) sample shown in Fig. 2 (c). This underlines the large mean free path of the electrons in insulating materials excited by extremely low photon energy which must be in the range of at least 4 nm, i.e., the thickness of the 20 ML MgO film. The small spectroscopic discrepancies between the free Co_2_MnSi surface and the buried interfaces originate solely from interface spectra smearing due to both quasi-elastic and inelastic scattering at defects directly at the interface. These defects are inevitably induced by the finite lattice mismatch between Co_2_MnSi and MgO^33^. Besides, these disloscations lead to a thickness-dependent surface defect density of the MgO layer and thus to MgO thickness-dependent work functions, as reported in Ref. 34 and visible in Fig. 2.

**Figure 3:**
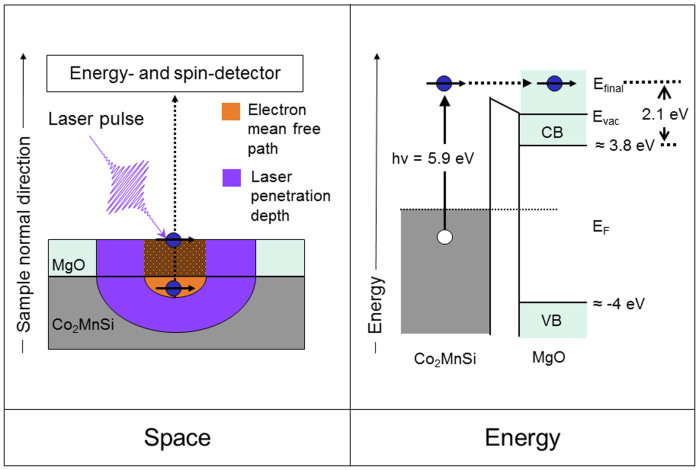
Conceptual principle of the ELEPS experiments. Left: The sample, constituted of a Co_2_MnSi thin film covered with MgO, is illuminated with pulsed laser light with photon energy *hν* = 5.9 eV. MgO is almost transparent to the laser, while the laser penetration depth (blue shaded area) in Co_2_MnSi is much larger than the inelastic mean free path of the electrons excited by the laser (orange shaded area). Moreover, the inelastic mean free path of the photoelectrons in MgO is virtually infinite (orange area with black pattern). As a consequence, photoelectrons originate mainly from the Co_2_MnSi/MgO interface and can travel without inelastic scattering in the MgO layer. The long inelastic mean free path of the photoelectrons in MgO is due to the fact that their excess energy is at maximum 2.1 eV above the conduction band minimum, which drastically reduces the phase space available for inelastic scattering. Right: Energy level alignment at the Co_2_MnSi/MgO system. The position of the valence band (VB) onset is extracted from the UPS measurements in Fig. 2 (a), while the position of the conduction band (CB) is inferred from the value of 7.8 eV of the MgO band gap taken from Refs. 10, 35. *E_vac_* = 3.9 eV for the Co_2_MnSi/MgO(10 ML) system.

Let us now explain the origin of the remarkable interface sensitivity. Figure 3 illustrates the principle of ELEPS. If applied to metals, this method is strongly surface sensitive^27^ and hence comparable to conventional UPS. If applied to a metal/insulator system like Co_2_MnSi/MgO, the high penetration depth of the used laser wavelength in MgO results in an excitation of electrons near *E_F_* in the uppermost metallic (Co_2_MnSi) layers which will traverse the metal/insulator interface. Inside the insulator (MgO) these electrons will transiently occupy states in the conduction band until reaching the surface and getting finally photoemitted. In the exemplary case of MgO, we extract from the UPS spectra in Fig. 2 the valence band onset at −4 eV. Assuming a band gap of 7.8 eV^10^,^35^ we can infer the conduction band minimum of MgO at a binding energy of 3.8 eV above *E_F_*. Since the excitation energy is 5.9 eV, only the lowest 2.1 eV of the conduction band will be accessible to the traversing electrons. In contrast to conventional UPS with excitation energies higher than 20 eV, these electrons will not be able to scatter inelastically with the MgO valence band electrons (in general, this process is the actual reason for the high surface sensitivity of UPS), since no final states for the potential scattering partners can be found: even for a maximum possible energy exchange of 5.9 eV, the MgO band gap is still larger and hence prohibits inelastic scattering. This is the ultimate reason for the extremely large mean free path of low energy electrons in insulating materials, translating in the interface sensitivity of the ELEPS method at FM/insulator interfaces.

### Electronic properties of the Co_2_MnSi/MgO interface

After we have provided experimental evidence for the high interface sensitivity of ELEPS, we now turn to the discussion of the electronic and spintronic properties of the Co_2_MnSi/MgO interface. A close inspection of the spectra in Fig. 1 and Fig. 2 (b) shows that the Δ_1_ minority surface state does not convert into an interface state when the Co_2_MnSi/MgO interface is formed. If this would be the case, like e.g. at the CoFe/MgO interface^7^, the resulting interface state should again lead to a distinct feature in the minority spectra and hence to a negative spin polarization near *E_F_* when p-polarized light is used. Since no additional features appear in the interface spectra compared to the free surface, we can thus infer that no interface states with either Δ_1_ or Δ_5_ symmetry are formed at the Co_2_MnSi/MgO interface in the investigated energy range. Although such interface states are predicted theoretically at *E_F_*^28^,^36^, they do not cross the Fermi level near the Γ point^37^. This explains not only the shape of our photoemission spectra, but also the high tunneling magneto resistance ratios obtained with CMS/MgO-based MTJs at low temperatures. In fact, Δ_1_ states contribute most to the tunneling current of such devices^38^, and the presence of minority states with Δ_1_ symmetry in the band gap would significantly reduce the MTJs performance.

### Wave function symmetries at the interface

**Figure 4:**
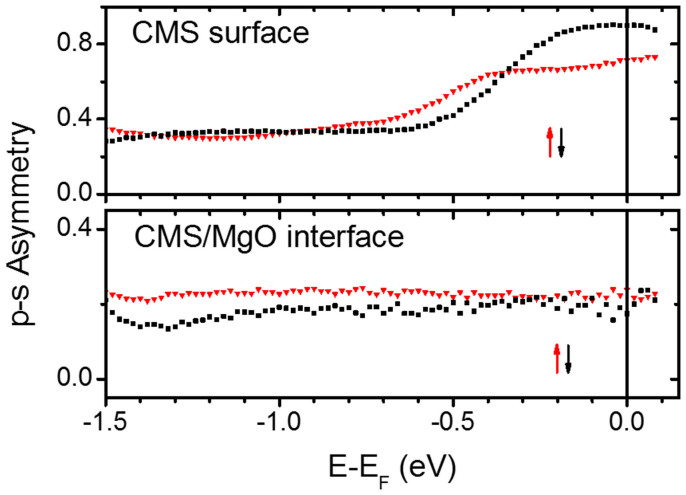
Spin-resolved interface wave function symmetry. Spin- and energy-resolved p-s asymmetry of the Co_2_MnSi free surface (upper panel) and the Co_2_MnSi/MgO(10 ML) interface (lower panel). The majority (minority) channel is marked with red triangles (black squares).

Here we evaluate the energy-dependent p-s asymmetry, defined as: 
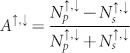
; where 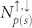 are the spin- and energy-resolved photoemission intensities recorded respectively for p(s)-polarized light. In Fig. 4 we plot the asymmetry of the minority (↓) and majority (↑) electrons for both the Co_2_MnSi surface (upper panel) and the Co_2_MnSi/MgO(10 ML) interface (lower panel). *A*^↑,↓^ allows to determine the relative spin-resolved Δ_1_/Δ_5_ contributions of the electronic wave functions probed by the photoemission process^27^. In fact, in our experimental set-up p-polarized light probes both Δ_1_ and Δ_5_ electronic states, while s-polarized light only probes Δ_5_ states. Thus, an increase of the p-s asymmetry at a certain energetic position means that additional Δ_1_ states contribute to the spectra at this energy. At the free Co_2_MnSi surface the majority channel shows enhanced asymmetry values near *E_F_* compared to higher binding energies due to majority surface resonances exhibiting Δ_1_-like symmetry, in line with recent spin-resolved UPS experiments^18^. The increase is even stronger in the minority channel, since the 5.9 eV photons additionally excite resonantly a minority surface state. At the Co_2_MnSi/MgO interface, the majority channel exhibits an almost energy-independent asymmetry, as expected from bulk band structure calculations^28^. Crucially, the minority channel shows a very similar behavior. This finding can be explained by temperature-related effects, as we discuss in the following.

We first recall that the strong temperature dependence of the spin polarization of the Co_2_MnSi/MgO interface - that is assumed in order to explain the lowered performance of Co_2_MnSi/MgO-based MTJs at room temperature- can be ascribed theoretically to two competing mechanisms (c.f. Fig. 1 (a)): The formation of new minority states inside the gap^21^,^22^,^23^,^24^ as well as the mirroring of the majority density of states into the minority channel. The latter process is assumed to be only marginal in the bulk of half-metals^24^, however latest theoretical considerations regarding the Co_2_MnSi/MgO interface found a distinctively weakened exchange coupling and hence non-collinear alignment of atomic interface moments at elevated temperatures, causing a significant TMR ratio decrease compared to Fe/MgO-based MTJs^26^. The latter process leads to a projection of majority states onto the minority band gap within the interface region, and hence will influence the spin-resolved ELEPS spectra. In fact, this scenario agrees perfectly with our observation that both the minority and majority channel possess almost an identical spectral shape as well as the same Δ_1_/Δ_5_ asymmetry near *E_F_*. These findings are furthermore in accordance with other experiments focused on temperature-dependent measurements at Co_2_MnSi^39^,^40^. Please note that bulk-sensitive magnetization measurements can not reveal this process, since only the interface magnetic moments are affected. The formation of other temperature-induced minority gap states, on the other hand, can be ruled out, as such states would posses a different Δ_1_/Δ_5_ asymmetry than the majority states present in the energetic region of the band gap.

Before concluding, we note that at the buried Co_2_MnSi/MgO interface, surface contributions are suppressed by the MgO top layer, and no further interface states are formed. The interface electronic structure thus resembles the Co_2_MnSi bulk properties, and hence the measured interface spin polarization is in qualitative agreement with the bulk photoemission calculations (including correlation effects) of Ref. 18.

## Conclusions

On the example of the bilayer Co_2_MnSi/MgO, we were able to study the electronic spin- and symmetry properties of a buried ferromagnetic/insulator interface by spin-resolved ELEPS. Even for insulator thicknesses of 4 nm, the electronic fingerprint of the free ferromagnetic surface is almost unchanged in the ELEPS experiment. This proves the sensitivity of ELEPS for interfaces buried below insulating materials. We applied this novel technique in order to reveal the fundamental properties of the very same buried Co_2_MnSi/MgO interface implemented in state-of-the art Heusler-based magnetic tunnel junctions. We found a complete suppression of the Δ_1_ minority surface state present at the Co_2_MnSi surface (whose presence would be detrimental for MTJs), and no indication for the formation of additional interface states. A detailed analysis of the spectral dependence, spin polarization and wave function symmetry of the interface-related photoemission spectra allowed us to rule out the presence of several temperature-induced minority states in the band gap, while both thermally activated non-collinear interface spin moments and photoemission related effects can explain our experimental data.

## Methods

### Sample fabrication and preparation

Four samples with the stacking structure 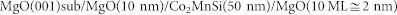 were fabricated^17^ to investigate the electron wavefunction symmetry and spin polarization of the Co_2_MnSi/MgO interface as well as the free Co_2_MnSi surface. Additionally Mn-rich 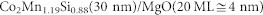 samples^16^ served to prove the truly interface-sensitive property of ELEPS. In all cases both epitaxial Co_2_MnSi and MgO grew as (001) layers with preferential L2_1_ crystal structure, i.e. highest ordering^16^,^17^. Crystalline structure and chemical composition of the Co_2_MnSi/MgO bilayers as well as the Co_2_MnSi surface were controlled by low energy electron diffraction and Auger electron spectroscopy. In agreement with previous investigations^34^, the samples showed high structural order and stoichiometric composition. Auger electron spectroscopy was further used to ensure the complete removal of the MgO top layer by gentle 500 eV *Ar*^+^ ion sputtering, performed at the Co_2_MnSi/MgO(10 ML) samples in order to investigate the bare Co_2_MnSi surface. A very short *Ar*^+^ ion sputtering cycle with a removal of max. 1 ML of MgO was also applied prior to photoemission experiments on the Co_2_MnSi/MgO(10 ML) system to remove residual carbon contaminations from the MgO surface. As expected, preferential sputtering at MgO did not take place^41^. Since the Co_2_MnSi/MgO(20 ML) sample showed in the Auger spectra only marginal carbon adsorption at the MgO surface due to less surface defects^34^, it was not *Ar*^+^ ion sputtered. As the final step before the photoemission experiments, all the samples were annealed to 450°C for at least 30 minutes. Hence, the interfaces studied in this work are identical to the one present in state-of-the-art devices fabricated by some of the authors^16^,^17^. All four samples with stoichiometric Co_2_MnSi showed identical interface spin polarizations and wave function asymmetries. For three of the samples, the MgO top layer was removed to study the free Co_2_MnSi surface, with fully reproducible results.

### Photoemission experiments

For the spin-resolved ELEPS experiments we used the fourth harmonic of a femtosecond Ti:Sa oscillator with photon energy *hν* = 5.9 *eV* as the light source^42^. A 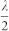 plate allows to switch between p- and s-polarization of the laser beam, which illuminates the sample under a degree of 45°. Because of the optical dipole selection rules^43^,^44^, s-polarized light excites only electrons with Δ_5_ symmetry at the Co_2_MnSi(100) surface, while p-polarized light probes both Δ_1_ and Δ_5_ states in case of an incoming beam angle of 45°^7^,^27^. For comparison with standard photoemission methods (performed typically with higher photon energies), spin-resolved ultraviolet photoemission spectroscopy (UPS) spectra were recorded using a commercial He VUV gas discharge lamp providing unpolarized light with *hν* = 21.2 *eV* (He I line). A spin detector (Omicron CSA-SPLEED) allows for energy- and spin-resolution of the photoemitted electrons. The energy resolution is set to 210 meV for ELEPS and decreased to 420 meV for UPS in order to compensate the lower photoemission yield. All measurements were conducted at room temperature in an ultrahigh vacuum chamber with a base pressure lower than 10^−10^ mbar. The sample surface normal points to the electron analyzer entry slit in [001] crystal direction, which is equivalent to the Γ – *X* direction of Co_2_MnSi in k-space. Hence all electrons originating from this part of the Brillouin zone are probed by our set-up. Furthermore, due to a finite detector acceptance angle of ±15° and an applied biasing voltage of −4 V, the Γ – *K* crystal direction is probed additionally by approx. 15% (at *hν* = 5.9 eV). Prior to the photoemission experiments, the samples were magnetized in remanence along the easy magnetization axis, i.e. the [110] crystal direction of Co_2_MnSi. The remanent magnetization of the samples, showing a squared hysteresis along the easy axis, is nearly 100% of the saturation magnetization^45^. The measured spin polarization is the projection of the spin polarization vector along this direction.

## Author Contributions

R.F., B.S., M.A. and M.C. wrote the main manuscript text. R.F. and M.C. prepared all figures. R.F. conducted all presented experiments. Y.O. and T.T. fabricated the investigated samples. H.N., M.O., Y.A., T.U. and M.Y. supervised sample fabrication, contributed to the respective methods section as well as to the discussion of the results. All authors reviewed the manuscript.
